# Mitochondrial dysfunction resulting from loss of cytochrome *c* impairs radiation-induced bystander effect

**DOI:** 10.1038/sj.bjc.6605087

**Published:** 2009-05-19

**Authors:** G Yang, L Wu, S Chen, L Zhu, P Huang, L Tong, Y Zhao, G Zhao, J Wang, T Mei, A Xu, Y Wang

**Affiliations:** 1 State Key Laboratory of Nuclear Physics and Technology, Peking University, Beijing 100871, People's Republic of China; 2 Key Laboratory of Ion Beam Bioengineering, Institute of Plasma Physics, Chinese Academy of Sciences, Hefei, Anhui 230031, People's Republic of China; 3 Shenzhen University, Shenzhen, Guangdong 518060, People's Republic of China

**Keywords:** radiation-induced bystander effect, mitochondrial dysfunction, cytochrome *c*

## Abstract

Cytochrome *c* is a pivotal protein that resides in mitochondria as component of mitochondria respiration and apoptosis initiator. Using murine cells lacking cytochrome *c*, we showed here that cytochrome *c*-deficient cells had attenuated reactive oxygen species/nitric oxide and micronuclei induction to radiation-induced bystander signals, indicating cytochrome *c* is essential for the bystander effect.

Mitochondria are pivotal cellular organelles for they not only generate the cellular ATP through oxidative phosphorylation but also initiate the intrinsic apoptosis (Li *et al*, 2000; Berg and Stryer, 2002). In recent years, emerging evidence indicated that mitochondria have also been implicated in radiation-induced bystander effect (RIBE) (Lyng *et al*, 2000, 2002; Limoli *et al*, 2003; Maguire *et al*, 2005; Murphy *et al*, 2005; Nugent *et al*, 2007; Tartier *et al*, 2007). Earlier, we reported that cells deficient in mitochondrial DNA (mtDNA) showed a significant reduction of bystander signalling (Chen *et al*, 2008). However, these studies are mainly based on mtDNA depletion, which many genes and related signalling pathways are deficient, or based on various pharmacological inhibitors to mitochondria function, which usually have nonspecific side effects (Mansfield *et al*, 2005). In addition, there might be random mutation of nuclear genes in generating the mtDNA-deficient cells treated with ethidium bromide for long period (King and Attardi, 1996; Mansfield *et al*, 2005). To further study the roles of mitochondria in RIBE, in this study, murine embryonic cells lacking cytochrome *c* (Cyt *c*) were used as a genetic model.

Cytochrome *c* resides in between the outer and inner membranes of mitochondria in living cells. It transports electrons from Complex III to Complex IV in the mitochondrial respiratory chain, crucial for synthesis of ATP through oxidative phosphorylation (Berg and Stryer, 2002). Loss of Cyt *c* prevents oxidation of cytochrome *c*1, keeping the Rieske iron-sulphur protein reduced (Berg and Stryer, 2002; Mansfield *et al*, 2005), and therefore causes mitochondrial respiration deficiency. Using this model, we provide genetic evidence that functional mitochondria are essential in the RIBE, and Cyt *c* is necessary for cells to respond radiation-induced bystander signals.

## Materials and methods

### Cell culture and co-culture system

Cytochrome *c*-normal (Cyt *c*^+/+^) and -null (Cyt *c*^−/−^) mouse embryonic fibroblast cell lines are kind gifts from Dr Xiaodong Wang. Genotyping was performed by PCR and cells were maintained as described earlier (Li *et al*, 2000). There was no significant difference in cellular morphology, reactive oxygen species (ROS) background and mitochondrial membrane potential between the Cyt *c*^+/+^ and Cyt *c*^−/−^ cells (Supplementary Figure 1). The doubling time for Cyt *c*^+/+^ and Cyt *c*^−/−^ cells was ∼16.8 and 20.8 h, respectively and the background of apoptosis was much higher in Cyt *c*^−/−^ cells than that of Cyt *c*^+/+^ cells as reported earlier (Supplementary Figure 2) (Li *et al*, 2000). The co-culture system was also described earlier (Zhu *et al*, 2008). Approximately, 4 × 10^4^ and 3 × 10^5^ cells for Cyt *c*-normal cells, as well as 6 × 10^4^ and 4.5 × 10^5^ cells for Cyt *c*-null cells, were seeded and cultured on inner and outer dishes in ∅35 and ∅60 mm culture dish separately. The culture medium was replaced every 24 h and 100% confluent culture was used for irradiation.

### Irradiation protocol

The parameters of the irradiation facility were described earlier (Zhu *et al*, 2008). In brief, the medium of outer and inner dishes were aspirated off before irradiation, and then the inner dishes were put into the outer dishes upside down quickly. At the same time, fresh medium was added, and the cells in the outer dish were irradiated immediately with 20 cGy dose of *α*-particles. After irradiation, the irradiated and bystander cells were co-cultured in the 37°C incubator, which was followed by micronuclei (MN), ROS and nitric oxide (NO) test.

### Micronuclei, ROS and NO test

For MN test, 3 h after irradiation, cells were trypsinised and prepared for micronucleus test using the cytokinesis-block technique as described (Fenech, 2000). The number of MN in at least 1000 binucleate cells was scored and the frequency of MN per 1000 binucleate cells was calculated.

For ROS and NO assay, medium transfer experiment was carried out as described (Chen *et al*, 2008). The 4-amino-5-methylamino-2′,7′-difluorofluorescein diacetate (DAF-FM diacetate) and 5-(and-6)-chloromethyl-2′,7′-dichlorodihydrofluorescein diacetate, acetyl ester (CM-H_2_DCFDA) (Molecular Probes, Eugene, OR, USA) were used to quantify the level of NO and ROS, respectively, as described (Wan *et al*, 2005; Chen *et al*, 2008). In brief, cells at confluency were stained with 5 *μ*M DAF-FM diacetate in Tyrode's solution or 2 *μ*M CM-H_2_DCFDA in D-Hanks for 30 min in incubator, and then washed twice with the proper buffer respectively. For the direct irradiation groups, the cells on the outer dishes were then irradiated with 20 cGy *α*-particles followed by 10 min incubation at 37°C. The fluorescence intensity was measured with a fluorescent reader (Spectra Max M2, Molecular Devices Sunnyvale, CA, USA. Excitation/emission: 495/515 nm for NO and ROS assay). For the irradiated cell conditioned medium (ICCM) treated groups, 100 *μ*l ICCM were added in each well of the 96-well microplate followed by 10 min incubation at 37°C, then assayed with the fluorescent reader. The fluorescence intensity was normalised to the sham-irradiated cultures. Statistical analysis was performed on the means of the data pooled from at least three independent experiments.

### Statistics

All data are pooled from at least three independent experiments, and the results are presented as means±s.d. Significance between two groups were assessed using Student's *t-*test. A *P*-value of <0.05 between groups was considered significant.

## Results

### Cyt *c*-null cells responded to radiation similar to Cyt *c*-normal cells

**Figure 1:**
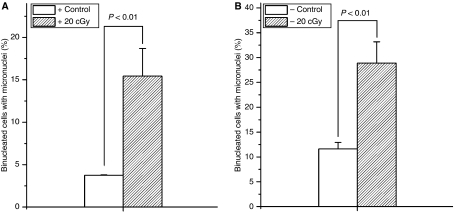
Micronuclei induction of Cyt *c*-normal (**A**) and Cyt *c*-null cells (**B**) irradiated with 20 cGy dose of *α*-particles. Data were pooled from at least three independent experiments, error bar=s.d.

As shown in Figure 1, with 20 cGy *α*-particles irradiation, the induction of MN in both Cyt *c*-normal and -null cells were increased significantly by ∼12 and ∼17%, respectively, of their corresponding controls. The results indicate that Cyt *c*-null cells could also respond to radiation-induced DNA damage. The different backgrounds in MN incidence for Cyt *c*-normal cells (3.75±0.05%) and Cyt *c*-null cells (11.61±1.31%) might reflect the important roles of Cyt *c* in maintaining genome stability (Cox and Hampton, 2007).

### Cyt *c*-null cells generated bystander signals after irradiation

**Figure 2:**
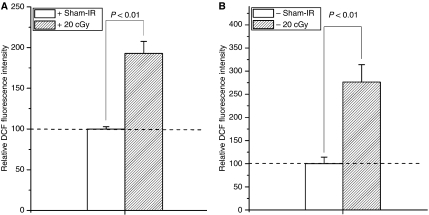
Fluorescence intensities of DCF were significantly increased in 20 cGy-irradiated Cyt *c*^+/+^ (**A**) and Cyt *c*^−/−^ cells (**B**). The fluorescence intensity was normalised to the sham-irradiated cultures. Data were pooled from at least three independent experiments, error bar=s.d.

**Figure 3:**
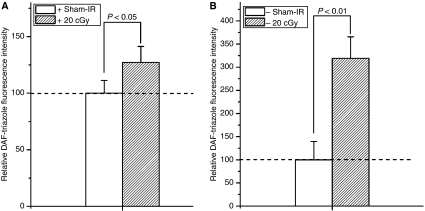
Fluorescence intensities of DAF-triazole were significantly increased in 20 cGy-irradiated Cyt *c*^+/+^ (**A**) and Cyt *c*^−/−^ cells (**B**). The fluorescence intensity was normalised to the sham-irradiated cultures. Data were pooled from at least three independent experiments, error bar=s.d.

As shown in Figures 2 and 3, after 20 cGy *α*-particles irradiation, the induction of ROS/NO in Cyt *c*-normal and -null cells increased 1.92/1.27 times (Figures 2A and 3A) and 2.76/3.18 times (Figures 2B and 3B) of their controls, respectively, indicating that Cyt *c*-null cells can also respond to irradiation in increase of the ROS and NO generations.

**Figure 4:**
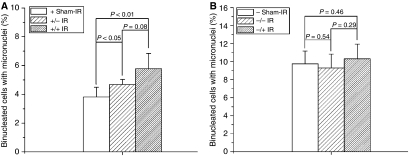
Micronuclei (MN) test. (**A**) Micronuclei induction of Cyt *c*-normal cells co-cultured with irradiated cells. + Sham-IR, Cyt *c*^+/+^ control; +/− IR, Cyt *c*^+/+^ cells co-cultured with irradiated Cyt *c*^−/−^ cells; +/+ IR, Cyt *c*^+/+^ cells co-cultured with irradiated Cyt *c*^+/+^ cells. (**B**) Micronuclei induction of Cyt *c*-null cells co-cultured with irradiated cells. − Sham-IR, Cyt *c*^−/−^ control; −/− IR, Cyt *c*^−/−^ cells co-cultured with irradiated Cyt *c*^−/−^ cells; −/+ IR, Cyt *c*^−/−^ cells co-cultured with irradiated Cyt *c*^+/+^ cells. Data were pooled from at least three independent experiments, error bar=s.d.

**Figure 5:**
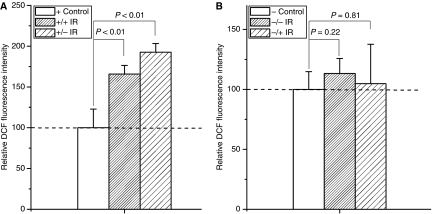
Fluorescence intensities of DCF were significantly increased in ICCM-treated Cyt *c*^+/+^ (**A**) but not in Cyt *c*^−/−^ cells (**B**). + Control, Cyt *c*^+/+^ control; +/− IR, Cyt *c*^+/+^ cells co-cultured with ICCM from irradiated Cyt *c*^−/−^ cells; +/+ IR, Cyt *c*^+/+^ cells co-cultured with ICCM from irradiated Cyt *c*^+/+^ cells. − Control, Cyt *c*^−/−^ control; −/− IR, Cyt *c*^−/−^ cells co-cultured with ICCM from irradiated Cyt *c*^−/−^ cells; −/+ IR, Cyt *c*^−/−^ cells co-cultured with ICCM from irradiated Cyt *c*^+/+^ cells. The fluorescence intensity was normalised to the control cultures. Data were pooled from at least 3 independent experiments, Error bar=s.d.

**Figure 6:**
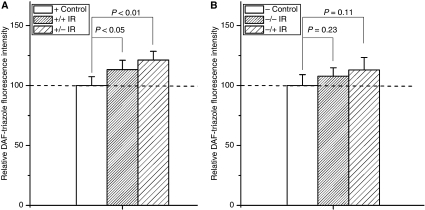
Fluorescence intensities of DAF-triazole were significantly increased in ICCM-treated Cyt *c*^+/+^ (**A**) but not in Cyt *c*^−/−^ cells (**B**). The fluorescence intensity was normalised to the control cultures. Data were pooled from at least three independent experiments, error bar=s.d.

When Cyt *c*-normal cells co-cultured with irradiated Cyt *c*-normal or -null cells, significant MN inductions were detected (5.78±1.06% for irradiated Cyt *c*-normal cells, *P*<0.01; 4.68±0.35% for irradiated Cyt *c*-null cells, *P*<0.05) (Figure 4A). In addition, as shown in Figures 5A and 6A, when Cyt *c*-normal cells co-cultured with ICCM from irradiated Cyt *c*-normal or Cyt *c*-null cells, significant induction of ROS/NO were also detected (Figure 5A for ROS, 165.77±10.76% for ICCM from Cyt *c*-normal cells, *P*<0.01; 192.59±10.72% for ICCM from irradiated Cyt *c*-null cells, *P*<0.01; Figure 6A for NO, 113.21±7.91% for ICCM from Cyt *c*-normal cells, *P*<0.05; 121.29±7.27% for ICCM from irradiated Cyt *c*-null cells, *P*<0.01). These results suggested that both Cyt *c*-normal and -null cells generated the bystander signals in response to irradiation.

### Cyt *c*-null cells attenuated the response to the radiation-induced bystander signals

As shown in Figure 4B, when Cyt *c*-null cells co-cultured with irradiated Cyt *c*-normal or -null cells, no significant induction of MN were detected (10.31±1.63% for irradiated Cyt *c*-normal cells, *P*=0.46; 9.3±1.53% for irradiated Cyt *c*-null cells, *P*=0.54), suggesting that Cyt *c*-null cells attenuated the response to radiation-induced bystander signals in the co-culture system and Cyt *c* is necessary for the proper sensing of bystander signals.

In addition, as shown in Figures 5B and 6B, when Cyt *c*-null cells co-cultured with ICCM from irradiated Cyt *c*-normal or Cyt *c*-null cells, no significant induction of ROS/NO were detected (Figure 5B for ROS, 104.65±33.98% for ICCM from Cyt *c*-normal cells, *P*=0.81; 113.25±12.63% for ICCM from irradiated Cyt *c*-null cells, *P*=0.22; Figure 6B for NO, 112.96±10.39% for ICCM from Cyt *c*-normal cells, *P*=0.11; 107.71±7.11% for ICCM from irradiated Cyt *c*-null cells, *P*=0.23). The results suggested that, compared with Cyt *c*-normal cells, Cyt *c*-null cells cannot respond to the ICCM and Cyt *c* might be necessary for the proper sensing of bystander signals.

## Discussion

Recent studies indicated that irradiated mtDNA-depletion cells are capable of generating bystander signals with less efficiency (Zhou *et al*, 2008). This study, together with several other studies (Lyng *et al*, 2000, 2002; Limoli *et al*, 2003; Maguire *et al*, 2005; Murphy *et al*, 2005; Nugent *et al*, 2007; Tartier *et al*, 2007; Chen *et al*, 2008), suggested that mitochondria represent the sensor of radiation-induced ionisation events. In this study, we showed that Cyt *c*-null cells can respond to radiation normally in ROS/NO generations as Cyt *c*-normal cells (Figures 2B and 3B). It was reported that NF-*κ*B/COX-2/PGE2 and NF-*κ*B/iNOS/NO pathways are critical to the RIBE (Hei *et al*, 2008; Zhou *et al*, 2008) and in Cyt *c*-null cells, NF-*κ*B, PI3K/Akt, and JNK are with similar kinetics to Cyt *c*-normal cells following exposure to UV irradiations (Li *et al*, 2000). Thus, the capability of Cyt *c*-null cells in RIBE induction (Mikkelsen and Wardman, 2003) might be due to its capability in the ROS/NO induction after irradiation. Earlier studies showed that irradiated mtDNA-depletion cells have lower efficiency in RIBE induction (Chen *et al*, 2008; Zhou *et al*, 2008). This phenomenon might partly be due to the less efficiency of the initial signal propagations based on functional mitochondria (Mikkelsen and Wardman, 2003). In this study, although co-cultured with irradiated Cyt *c*-null cells could also induce significant MN incidence in Cyt *c*-normal cells (Figure 4A, *P*<0.05), the average MN induction efficiency was lower than that of cells co-cultured with irradiated Cyt *c*-normal cells (Figure 4A). However, similar NO induction but higher ROS induction were detected in Cyt *c*-normal cells treated with ICCM from irradiated Cyt *c*-null cells, indicating other signals except ROS/NO might also contribute to the bystander effect.

In bystander cells, earlier studies showed that functional mitochondria play important roles in responding the extracellular bystander signals (Murphy *et al*, 2005; Nugent *et al*, 2007; Zhou *et al*, 2008). In this study, the results suggested that Cyt *c*-null cells attenuate the response to radiation-induced bystander signals in the generation of ROS/NO and MN induction (Figures 4B, 5B and 6B), suggesting that Cyt *c* or mitochondrial function is required for responding the bystander signals. It was proposed that mtDNA-depleted cells thus deficient in mitochondrial respiration did not show a radiation-stimulated increase in ROS/RNS (Mikkelsen and Wardman, 2003). It was also suggested that Cyt *c*-null cells could not sufficiently increase mtROS production as Cyt *c*-normal cells upon hypoxic exposure (Mansfield *et al*, 2005). In this study, no significant induction of ROS/NO generations were detected in the Cyt *c*-null cells treated with ICCM from either irradiated Cyt *c*-normal or -null cells, whereas significant induction of ROS/NO generations were detected in the ICCM-treated Cyt *c*-normal cells, indicating Cyt *c*-null cells attenuate the response to the bystander signals. The disability to induce the ROS/NO suggested that Cyt *c*-null cells cannot effectively stimulate the downstream signalling processes in response to the bystander signals. Alternatively, Cyt *c*-null cells was reported to show increased sensitivity to cell death signals triggered by TNF-*α* (Li *et al*, 2000). Cytokines including TNF-*α* has long been suggested to play important roles in RIBE (Hei *et al*, 2008). The bystander Cyt *c*-null cells, which receive the signals, might have less survival due to the increased sensitivity and its high background MN incidence.
