# Supplementary material for: Mitochondrial dysfunction resulting from loss of cytochrome c impairs radiation-induced bystander effect
Source: Br J Cancer. 2009 May 19;100(12):1912–6. doi: 10.1038/sj.bjc.6605087 (PMC2714242; doi:10.1038/sj.bjc.6605087)
Supplement: Supplementary Figures 1 and 2 [file 6605087x1.doc]

**Supplementary data**


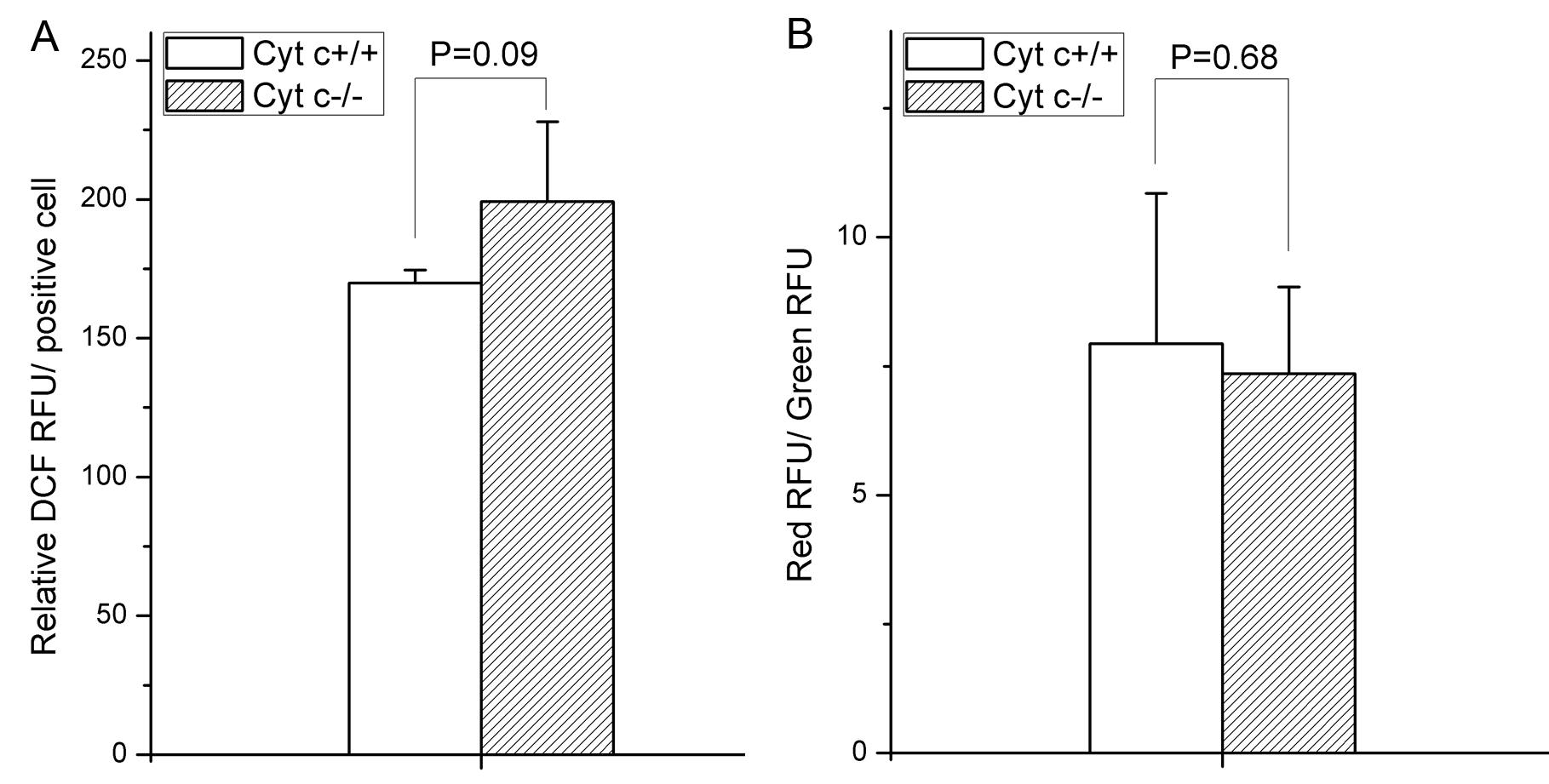


Supplementary Fig 1 the background ROS (A) and mitochondria membrane potential (B) in the cyt c+/+ and cyt c-/- cells. Data were pooled from at least 3 independent experiments, Error bar=SD. ROS level was assayed with flow cytometry using CM-H_2_DCFDA (5-(and-6)-chloromethyl-2′,7′-dichlorodihydrofluorescein diacetate, acetyl ester, Molecular Probes, Eugene, Oregon, USA) fluorescence, while the mitochondrial membrane potential was detected with JC-1 (5, 5’, 6, 6’-tetrachloro-1, 1’, 3, 3’- tetraethylbenzimidazolylcarbocyanine iodide, Molecular Probes, Eugene, Oregon, USA) assay through a flurescence reader.


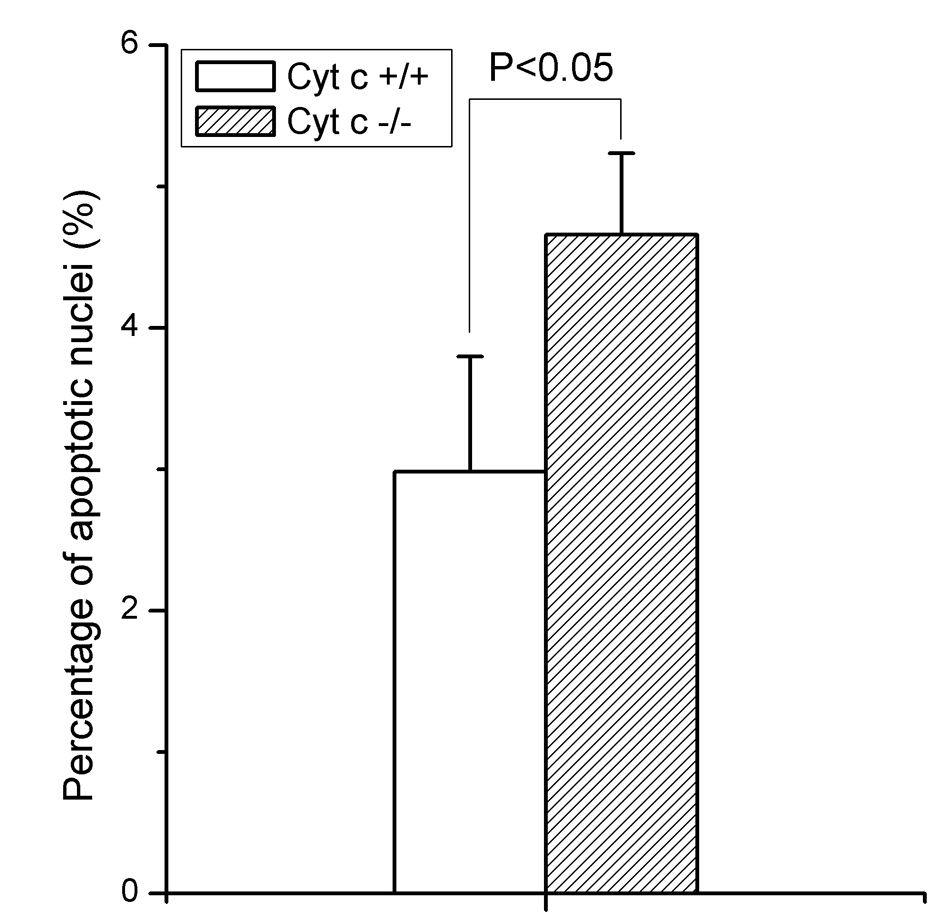


Supplementary Fig 2 background apoptosis in cyt c+/+ and cyt c-/- cells. Data were pooled from at least 3 independent experiments, Error bar=SD. Apoptotic nuclei were stained with DAPI (4',6-diamidino-2-phenylindole, dihydrochloride, Molecular Probes, Eugene, Oregon, USA) .
